# Better social reversal learning is associated with a more social approach across time

**DOI:** 10.1038/s41598-024-58348-5

**Published:** 2024-04-10

**Authors:** Reut Zabag, Yogev Kivity, Eva Gilboa-Schechtman, Einat Levy-Gigi

**Affiliations:** 1 Department of Psychology, Bar-Ilan University, Ramat Gan, Israel; 2 Department of Psychology, Yale University, New Haven, CT USA; 3 Gonda Multidisciplinary Brain Center, Bar-Ilan University, Ramat Gan, Israel; 4 Faculty of Education, Bar-Ilan University, Ramat Gan, Israel

**Keywords:** Social anxiety, Cognitive flexibility, Belief updating, Social approach, Longitudinal design, Psychology, Human behaviour

## Abstract

Flexibly updating behaviors towards others is crucial for adaptive social functioning. Previous studies have found that difficulties in flexibly updating behaviors are associated with social anxiety (SA). However, it is unclear whether such difficulties relate to actual social behaviors. The current study investigated the relationships between negative-to-positive social reversal learning, social approach behavior, and SA across time. Participants (MTurk, Time 1 = 275, Time 2 = 126, 16 weeks later) completed a performance-based social reversal-learning task. In the initial phase, participants learned that interactions with certain individuals are associated with negative outcomes, whereas interactions with other individuals are associated with positive outcomes. In the reversal phase, these associations were reversed, requiring participants to update their behaviors. The relationships between the performance in the task, SA severity, and social approach behavior reported by participants were assessed cross-sectionally and longitudinally. We found that negative-to-positive updating was negatively associated with SA severity. Furthermore, negative-to-positive updating was positively correlated with social approach behavior, both cross-sectionally and prospectively. Hence, individuals with better negative-to-positive updating at Time 1 reported significantly more social approach behaviors across time. The results support the role of negative-to-positive updating as a mechanism associated with SA and social approach, advancing and refining interpersonal and cognitive theories of SA.

## Introduction

*“Progress is impossible without change, and those who cannot change their minds cannot change anything.”*

*George Bernard Shaw.*

In today’s rapidly changing social world, the ability to change beliefs and behaviors toward other people is more critical than ever. Indeed, cognitive flexibility, the ability to update representations and behaviors based on external or internal demands, was suggested and found to be important for well-being and mental health^1,2^. Recent research suggests that psychopathology and specifically social anxiety (SA) are associated with difficulties in cognitive flexibility^3–7^.

SA is a common mental health condition characterized by an excessive fear of social situations and concerns regarding being scrutinized or judged by others^8^. Approximately one in nine individuals will struggle with SA disorder at some point in their lives^9^. SA significantly impacts a person’s daily life and manifests in various ways, including fear of public speaking, holding negative beliefs about the self, and avoidance of parties or other social events^10–13^. Even subclinical levels of SA are linked to avoidance of social situations or intense distress when faced with such situations^10^. Social withdrawal (avoidance of social events and interpersonal interactions) is highly associated with SA^14^ and is also postulated to maintain it.

Recent theories and research converge in proposing that SA is associated with deficits in cognitive flexibility^1^. SA was found to be associated with difficulty in positively updating initially negative interpretations^3,4,6^. Similarly, SA was associated with more avoidance behavior toward a previously punishing avatar, irrespective of their subsequent positive behavior^15^. Finally, recent studies have further supported that SA is associated with a struggle to update an initially negative impression of another person in the light of new positive information^16–18^. These deficits appear to be selective to social context^17,19^. Those impairments were also found to be specific to positively updating negative information and absent in negatively updating positive information^3–6,15,16^.

Cognitive flexibility was found to be linked to other aspects of well-being^20^. For example, better cognitive flexibility was associated with less rumination and more efficient cognitive reappraisal^21,22^. In addition, participants who did not suffer from deficits in belief updating reported having more friends^23^. Recently, difficulties in positive updating were found to be associated with more self-reported social withdrawals^24,25^. While important, these links between updating and social functioning did not focus on social behaviors and have been assessed only cross-sectionally. The present study aimed to shed light on one of the mechanisms underlying social functioning and SA maintenance by testing whether deficits in updating are associated with social approach behaviors cross-sectionally and longitudinally.

We examined cognitive flexibility, social approach, and SA in an ecological-longitudinal design. First, we aimed to further test whether SA is associated with difficulty in positively updating negative information about others (*inflexible positive updating in SA*). Second, we predicted that better performance in negative-to-positive updating is associated with more social approach concurrently and longitudinally (*social approach facilitation by positive updating*). Third, we postulated that SA is associated with fewer social approaches (*diminished social approach in SA*).

## Methods

### Power analysis

Sample sizes were calculated using the G*Power software^26^. Based on effect sizes found in previous studies^17,25^, we expected to observe a small-sized effect for all hypotheses (Cohen’s f = 0.16). A-priori power analysis for mixed factor repeated measures Generalized Linear Model (GLM) was conducted to detect this effect size with a significance (α) of 5% and power (1 − β) of 80%. This analysis suggested the need to recruit at least 203 participants.

### Participants

We recruited 381 participants through Amazon’s Mechanical Turk (MTurk). MTurk provides an online crowdsourcing platform with access to large and diverse samples^27^. Inclusion criteria for the study were: 18 years or older, being a resident of the United States, and high-quality performance on previous MTurk tasks (i.e., an acceptance ratio ≥ 95%). Participants were excluded due to (a) performance suggestive of fraudulent internet use, such as completing the study from an IP address identical to other study participants (n = 87); (b) performance indicative of low conscientiousness (filling all items of the questionnaires, including the reversed items, with zero standard deviation; very short duration of survey completion; failing an attention check; n = 19). In addition, participants who did not follow the initial instructions were automatically excluded and could not continue the task. A total of 275 participants were included in the final analyses of Time 1 and, out of them, 126 participants took part in Time 2 (see^25,28^ for similar data cleaning procedures). Participants who took part in both Time 1 and Time 2 were older and had significantly lower levels of SA compared to those who participated only in Time 1. All demographic characteristics of the participants are presented in Table 1.

**Table 1:** Frequencies or means and standard deviations (in parentheses) of demographic characteristics, psychopathology severity, task performance parameters and social approach behavior.

Variable	Cronbach’s alpha	Mean/frequencies	SD
Time 1 (n = 275):			
Demographic characteristics			
Gender (percentage of females)		51.6	
Age		38.91***	11.94
Education		15.51	2.20
Ethnicity (in percentage)			
Caucasians		79.6	
African Americans		6.2	
Hispanics		5.1	
Asians		6.9	
Native Americans		0.4	
Other		1.8	
Psychopathology severity			
LSAS	0.971	47.33*	29.50
SPIN	0.957	20.81*	16.55
Percentage of individuals with social anxiety scores above clinical cutoff (LSAS > 50; SPIN > 20)		36.73	
Performance parameters (in percentage)			
Total engagement (approach) decisions		47.63	12.61
Overall accuracy in negative-outcome associations learning	0.838	76.69	13.74
Overall accuracy in positive-outcome associations learning	0.920	62.43	20.65
Overall accuracy in positive-to-negative updating	0.807	81.56	17.01
Overall accuracy in negative-to-positive updating	0.958	72.68	32.93
Social approach behavior		0.86	0.99
Time 2 (n = 126):			
Demographic Characteristics			
Gender (% females)		49.2	
Age		42.18***	12.09
Education		15.53	2.01
Psychopathology severity			
LSAS		41.35*	28.21
SPIN		17.63*	14.78
Social approach behavior		0.74	0.84

LSAS, Liebowitz social anxiety scale; SPIN, social phobia inventory.

*p < 0.05, ***p < 0.001.

*Independent t-test results revealed that participants who took part in both Time 1 and Time 2 were older and had significantly lower levels of social anxiety compared to those who participated only in Time 1. Overall accuracy in negative-outcome associations learning = percentage of avoidance decisions to a negative outcome “people” in the learning phase. Overall accuracy in positive-outcome associations learning = percentage of approach decisions to a positive outcome “people” in the learning phase. Overall accuracy in positive-to-negative updating = percentage of avoidance decisions to a negative outcome “people” in the updating phase that were associated with positive outcome during the learning phase. Overall accuracy in negative-to-positive updating = percentage of approach decisions to a positive outcome “people” in the updating phase that were associated with negative outcome during the learning phase. Social approach behavior = number of social events attended as reported by the participants.

### Measures

#### SA-severity

***Liebowitz Social Anxiety Scale*—*Self-Report version*** (LSAS-SR)^29^ is a 24-item scale that assesses levels of anxiety and avoidance in social or performance situations, using a 0–3 Likert-type scale (A Cronbach’s alpha of 0.971 was obtained in the current sample).

***Social Phobia Inventory*** (SPIN)^30^ is a 17-item self-report scale that assesses participants’ fear, rate of avoidance, and physiological discomfort in social situations. Each item is rated on a 0–4 Likert-type scale (A Cronbach’s alpha of 0.957 was obtained in the current sample).

SA was computed as the mean standardized scores of the LSAS and SPIN questionnaires (the correlation between LSAS and SPIN was r = 0.89, *p* < 0.001) in order to enhance the convergent validity and following previous studies^16,17^. A higher score indicates a greater severity of SA.

#### Social approach

Participants were asked: “Did you do anything social during the last weekend? (Thursday through Sunday)”. If they answered “yes,” they were asked—“In how many social events did you take part?” Their responses were recorded.

#### Social reversal learning task

**Figure 1:**
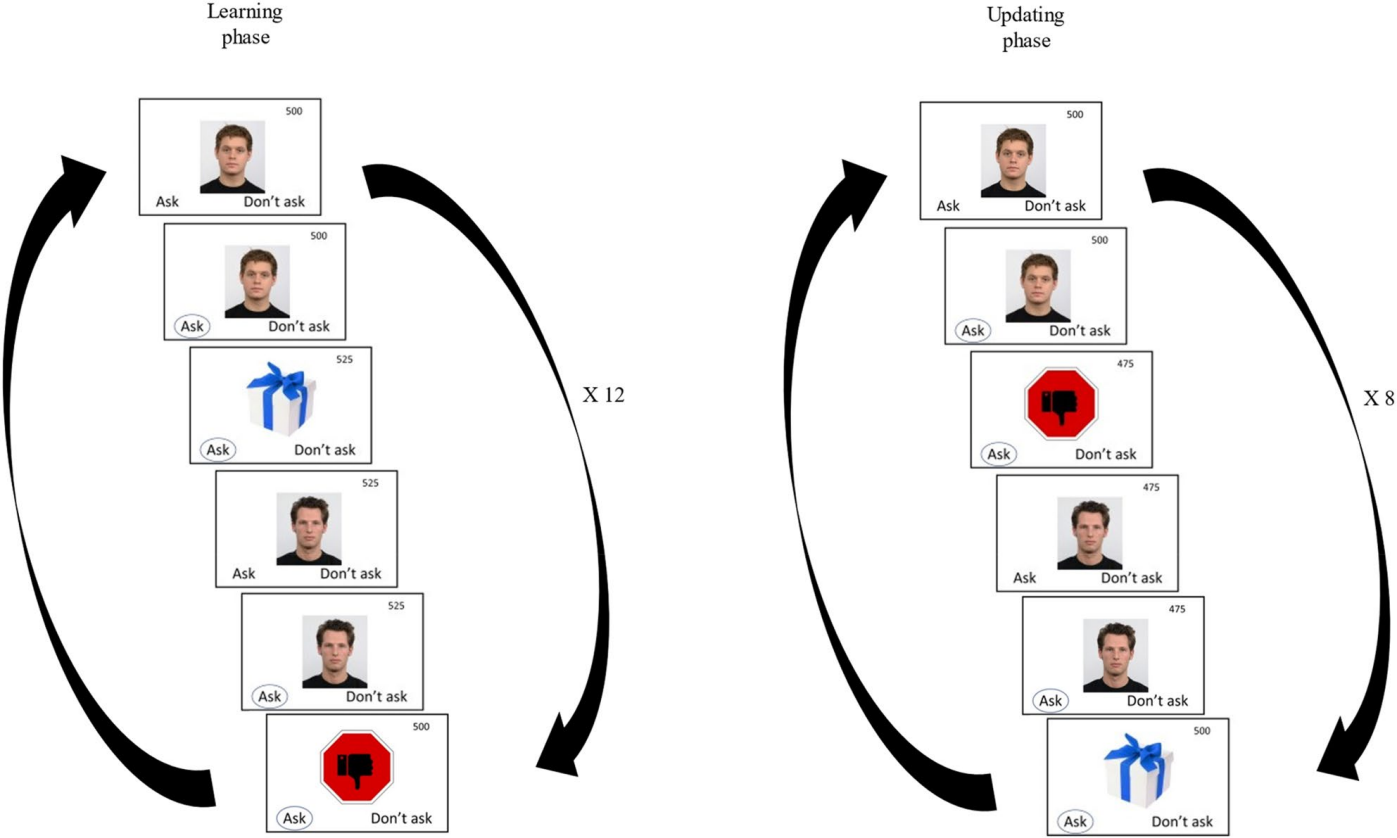
The social reversal learning task. A representation of the learning phase and the updating phase. In the learning phase, participants learned which ‘people’ provided positive feedback and which provided negative feedback. In the updating phase, the stimulus-outcome associations were changed. The outcome of the ‘people’ was reversed: some of those associated with a positive outcome in the learning phase became associated with a negative outcome in the updating phase, and vice versa. The pictures of the ‘people’ in the task are taken from the Radboud Faces Database^48^.

The well-validated task^16^ consists of two phases: learning and updating (reversal learning) (See Fig. 1). At the beginning of the task, participants were instructed that their aim was to maximize the points gained by deciding to interact (approach) or not interact (avoid) with one of eight “people” (for full instructions, see the link below). In each trial, an image of a person (a male with a neutral facial expression) was presented on the screen. Participants had to decide whether to approach or avoid this person. Approaching a person assigned to a positive outcome led to positive feedback (gift sign) and points gained, and approaching one assigned to a negative outcome led to negative feedback (stop sign) and points lost. Avoiding a person scored no points. During the learning phase, four “people” were assigned to a positive outcome and four negative outcomes. Participants learned which “people” to approach and which to avoid by trial and error. The learning phase consisted of 12 blocks of 8 stimuli each, resulting in 96 trials. A subsequent updating (reversal learning) phase was presented without any signaled cue or delay. In this phase, the stimulus-outcome associations were changed, and the outcome for six out of the eight “people” was reversed. Specifically, three of the four “people” associated with a positive outcome in the learning phase became associated with a negative outcome in the updating phase, and vice versa. Participants had the opportunity to change their behaviors and beliefs. The updating phase consisted of 8 blocks of 8 stimuli each, resulting in 64 trials. At the end of the task, participants were paid proportionally to the points they earned (up to $2). The task and its instructions can be found here:

https://barilanpsychology.qualtrics.com/jfe/form/SV_796KjwjKLwSpVfn

Performance and reliability measures are presented in Table 1.

### Procedure

All participants signed an informed consent after the nature of the process had been fully explained and before officially taking part in the study. Participants first completed the Social Reversal Learning Task and then filled out self-report questionnaires through a secure research software service (Qualtrics). At the end of the study, participants were debriefed and compensated for their participation. Sixteen weeks after the initial assessment, participants were contacted and asked about their social approach behavior.

The investigation was carried out in accordance with the latest version of the Declaration of Helsinki, and the Bar-Ilan University Ethics Committee approved the research.

## Results

Table 1 presents demographic characteristics, psychopathology severity, and performance parameters. Accuracy levels were calculated as the percentage of correct responses (i.e., decisions increasing the total gain in the task): an approach to positive outcome “people” and avoidance from negative outcome “people”. Negative stimulus-outcome learning represents the percentage of avoidance decisions to a negative outcome “people” in the learning phase. Positive stimulus-outcome learning represents the percentage of approach decisions to a positive outcome “people” in the learning phase. Positive-to-negative updating represents the percentage of avoidance decisions to a negative outcome “people” in the updating phase that were associated with positive outcome during the learning phase. Negative-to-positive updating represents the percentage of approach decisions to a positive outcome “people” in the updating phase that were associated with negative outcome during the learning phase. Because participants were not aware of the outcome of each person when they first saw them, we did not include the first response to each new person in the learning and updating phases in our analyses.

Table 2 presents the correlations between learning and updating accuracies, social approach, and SA. The correlations concerning the social approach were spearman due to the non-normal distribution of these measures. As can be seen from the Table, Accuracies during the updating phase were positively associated with each other and with accuracies during the learning phase. Negative-to-positive updating was adversely associated with SA and positively associated with the number of social events attended at Time 1 and Time 2. The number of social events attended at Time 1 was positively associated with the number of social events attended at Time 2.

**Table 2:** Pearson and Spearman correlations of social anxiety severity, accuracies during learning and updating phases, and social approach behavior.

Variable	1	2	3	4	5	6
1. Social anxiety	–					
2. Negative stimulus-outcome learning	0.06	–				
3. Positive stimulus-outcome learning	− 0.08	0.03	–			
4. Positive-to-negative updating	0.05	0.57***	0.31***	–		
5. Negative-to-positive updating	− 0.18**	0.14*	0.61***	0.29***	–	
6. Social approach behavior (Time 1)^a^	− 0.10^^^	0.04	0.08	− 0.03	0.17*	–
7. Social approach behavior (Time 2)^ab^	− 0.02	0.08	0.10	0.03	0.13***	0.41***

Negative-outcome associations learning = percentage of avoidance decisions to a negative outcome “people” in the learning phase. Positive-outcome associations learning = percentage of approach decisions to a positive outcome “people” in the learning phase. Positive-to-negative updating = percentage of avoidance decisions to a negative outcome “people” in the updating phase that were associated with a positive outcome during the learning phase. Negative-to-positive updating = percentage of approach decisions to a positive outcome “people” in the updating phase that were associated with a negative outcome during the learning phase. Social approach behavior = number of social events attended, as reported by the participants. Social anxiety = social anxiety levels as measured by the mean of standardized scores of the LSAS and SPIN.

LSAS, Liebowitz social anxiety scale; SPIN, social phobia inventory.

^^^*p* < 0.08, *p < 0.05, **p < 0.01, ***p < 0.001.

^a^Spearman correlations.

^b^N = 126.

**Figure 2:**
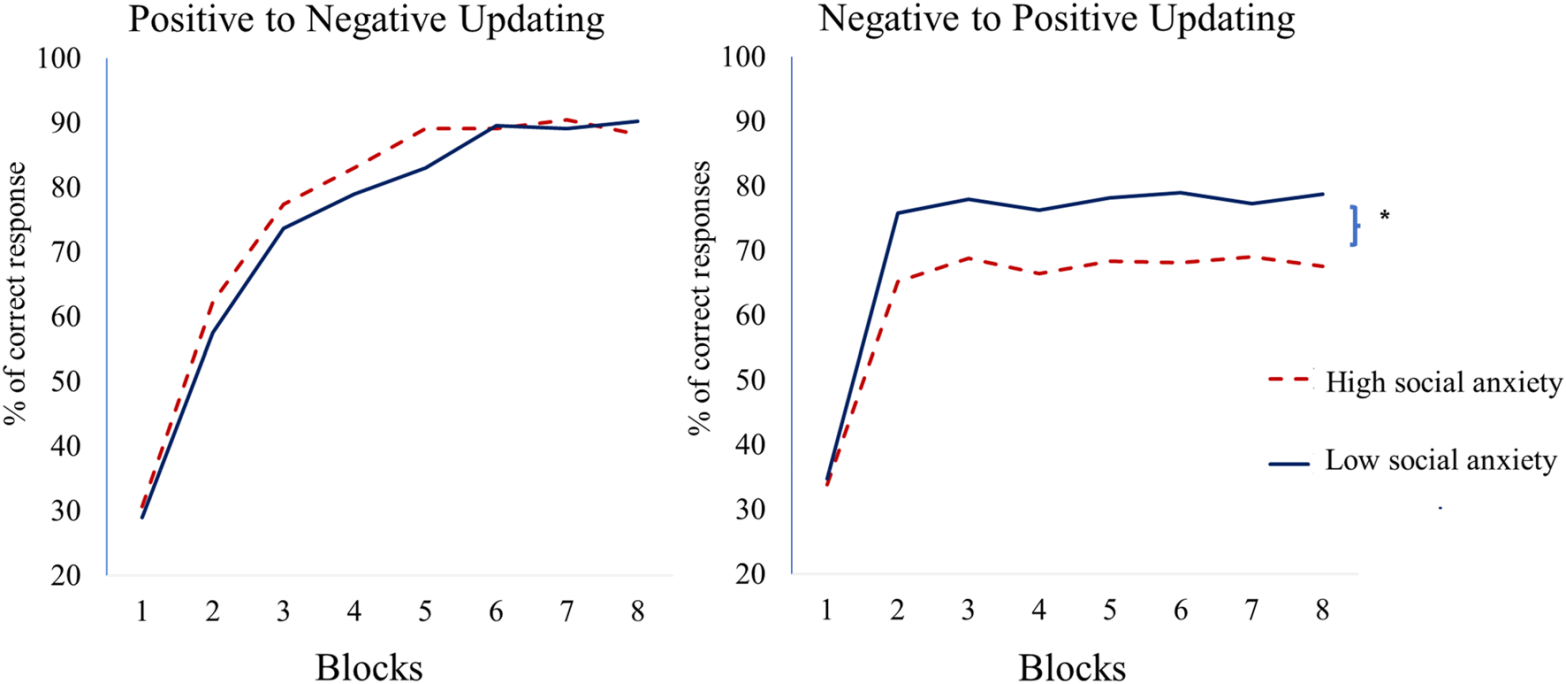
The percentage of correct responses (accuracy) during the updating phase of the social reversal learning task is depicted. The percentage of correct responses (accuracy) is presented on the Y-axis, while the task blocks (1–8) are displayed on the X-axis. We examined whether social anxiety is associated with a difficulty in positively updating negative information about others (inflexible positive updating in social anxiety). Results revealed that during the negative-to-positive updating phase, participants with higher levels of social anxiety (dashed red line) demonstrated lower accuracy compared to participants with lower levels of social anxiety (solid blue line). Conversely, in the positive-to-negative updating phase, no differences were found between participants with high and low social anxiety. *Note.* For simplicity, the figures are presented based on a median split of social anxiety symptoms.

To examine the “inflexible positive updating in SA” hypothesis, a repeated measures GLM was conducted on decision accuracy (i.e., the percentage of correct responses) in the updating-phase. Updating direction (positive-to-negative vs. negative-to-positive) and Block (1–8) were within-subject variables; SA (continuous) was a between-subject covariate variable. The full description of the findings is presented in Table 3. In the following, we review only the findings of our specific hypothesis. In line with our prediction, results revealed a significant two-way interaction between SA and Updating Direction (F (1, 273) = 9.66, *p* = 0.002, η2 = 0.034). A pictorial depiction of the findings appears in Fig. 2 (for simplicity, results are presented based on a median split of SA). To examine the source of the interaction, we conducted an identical GLM repeated measures analysis separately on negative-to-positive updating and positive-to-negative updating. Results revealed that SA was associated with reduced negative-to-positive updating (F (1, 273) = 7.58, *p* = 0.006, η2 = 0.027). However, SA was not associated with alterations in positive-to-negative updating (F (1, 273) = 3.12, *p* = 0.079). Results remain quantitatively identical after controlling for overall decision accuracy during the learning phase.

**Table 3:** The main effects and interactions of the generalized linear model on percentage of correct responses (accuracy) during the updating phase.

Effect	F	df	*p*-value	η2
Total updating				
Direction	11.42	1, 273	0.001	0.040
Social anxiety	3.04	1, 273	0.082	0.011
Block	325.02	1, 273	< 0.001	0.543
Social anxiety*direction	9.66	1, 273	0.002	0.034
Social anxiety*Block	2.81	1, 273	0.095	0.010
Direction*block	34.96	1, 273	< 0.001	0.114
Social anxiety*direction*block	1.92	1, 273	0.167	0.007
Negative-to-positive updating				
Social anxiety	7.58	1, 273	0.006	0.027
Block	135.83	1, 273	< 0.001	0.332
Social anxiety*block	4.52	1, 273	0.034	0.016
Positive-to-negative updating				
Social anxiety	3.12	1, 273	0.079	0.011
Block	225.42	1, 273	< 0.001	0.452
Social anxiety*block	0.90	1, 273	0.509	0.003

Direction = positive-to-negative updating or negative-to-positive updating. Block = the sequential progression of trials within the updating phase, from the beginning to the end. Social anxiety = social anxiety levels as measured by the mean of standardized scores of the LSAS and SPIN.

LSAS, Liebowitz social anxiety scale; SPIN, social phobia inventory.

To examine the “social approach facilitation by positive updating” hypothesis and the “diminished social engagement in SA” hypothesis, a Structural Equation Modeling (SEM) was employed on the participants who took part at both Time 1 and Time 2. WLS (weighted least squares) was used as an estimator because the assumptions of normality and equal variance were not met. Significant effects are indicated by 95% CIs that do not overlap with zero. The model was fit to the data using the lavaan package for R. Our model considered negative-to-positive updating and social approach at Time 1 as predicting social approach at Time 2. Note that alternative models were not supported by the data (see supplementary materials). This suggests that the hypothesized model is the most tenable model. The model displayed an excellent fit: χ2 (1, N = 126) = 0.603, *p* = 0.270. Root-Mean-Square Error of Approximation (RMSEA) = 0.00, the Confirmatory Fit Index (CFI) = 1.00, and the Tucker-Lewis index (TLI) = 1.236. Standardized root mean square residual (SRMR) = 0.013.

**Figure 3:**
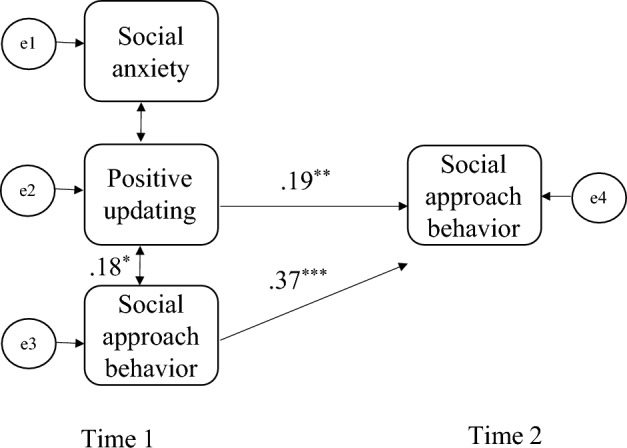
Structural equation modeling (SEM) of social anxiety severity, (negative-to-)positive updating accuracy, and social approach behavior at time 1 as predicting social approach behavior at time 2 (16 weeks later). Using the SEM, we examined whether better performance in negative-to-positive updating is associated with a more social approach behavior cross-sectionally and longitudinally (social approach facilitation by positive updating hypothesis). We also examined whether social anxiety is associated with less social approach behavior (diminished social approach in social anxiety hypothesis). *Note*. ^*^p < 0.05, ^**^ *p* < 0.01, ^***^ *p* < 0.001. Social anxiety = social anxiety levels as measured by the mean of standardized scores of the LSAS and SPIN. LSAS = Liebowitz social anxiety scale. SPIN = social phobia inventory. Positive updating = accuracy in the Social Reversal Learning Task as measured by the percentage of approach decisions to a positive outcome “people” in the updating phase that were associated with a negative outcome during the learning phase. Social approach behavior = number of social events attended, as reported by the participants.

The “social approach facilitation by positive updating hypothesis” was supported. We found that negative-to-positive updating was positively associated with social approach at Time 1 (*β* = 0.18, *SE* = 0.33, *p* = 0.036) and predicted social approach at Time 2 (*β* = 0.19, *SE* = 0.01, *p* = 0.001). Social approach at Time 1 predicted social approach at Time 2 (*β* = 0.37, *SE* = 0.08, *p* < 0.001). SA was not associated with social approach (*β* = − 0.04, *SE* = 0.13, *p* = 0.633). Thus, no support was found for the “diminished social engagement in SA hypothesis”. (See Fig. 3).

Full data and SEM analysis code are presented in https://osf.io/xhzrc/?view_only=e5e2f557355549f4a90180208a57f7d8.

## Discussion

The current longitudinal study assessed the association between the ability to positively change behaviors towards others, social approach behaviors, and SA. The results further support previous findings, suggesting an inverse association between negative-to-positive updating and SA severity. Most importantly, negative-to-positive updating was associated with social approach behaviors, both concurrently and longitudinally. This association was found above and beyond the impact of SA.

The results suggest that updating performance on a computer-based task is indeed associated with actual real-life behaviors. People who are able to change previously avoidant behavior when facing new positive information also report that they engaged in more social approach behaviors. On the other hand, people who are less likely to positively update their behaviors engaged in significantly fewer approach behaviors. It is possible that their updating difficulties result in ignoring new positive information about other people and hence limit their social world. Interestingly, research on children has found that better cognitive flexibility is associated with better social understanding^31^. Moreover, enhanced executive functioning performance, a construct close to cognitive flexibility, predicted improved theory of mind in a short longitudinal study^32,33^. Finally, cognitive flexibility also moderated the relationship between a supportive parenting style and prosocial behavior^34^. It is possible that these components mediate the association found in the current study.

Our results align with previous findings regarding cognitive flexibility, indicating that SA is associated with difficulty in positively updating avoidance behaviors toward others, negative beliefs about the self and others, as well as negative interpretations of social scenarios^4–6,16^. Inflexible behaviors and belief updating may contribute to the maintenance of SA symptoms, as individuals may become stuck in negative thought patterns and have difficulty seeing alternatives. For example, a person high in cognitive flexibility will notice that her unpleasant neighbor is becoming friendlier as time passes. This individual might re-approach her neighbor and start a conversation. By doing so, she is challenging her previously negative beliefs and possibly updating them. In contrast, an inflexible individual may fail to change their negative beliefs and behaviors and take this same approach, which could result in the maintenance or even worsening of their negative cognitions and affect. This person may be confirming their negative beliefs due to their tendency to dismiss disconfirming evidence, creating a self-reinforcing negative feedback loop. More broadly, an inflexible individual may also continue to engage in strategies that are no longer useful, such as maladaptive post-event processing, negative social expectations, or ineffective regulation^6,24^. As a result of comorbid difficulty in cognitive flexibility, high SA individuals may struggle to overcome social obstacles, such as rejection or exclusion, and maintain a negative view of themselves and their social world.

The current study’s findings may be viewed through the lens of SA and schemas. Previous research has suggested that SA is associated with schemas related to disconnection/rejection, other-directedness, and impaired autonomy and performance domains^35^. These early maladaptive schemas have been found to underlie and prospectively predict symptoms of SA^36^. It is plausible that these schemas are activated when individuals high in SA engage in negative social feedback during the task. For instance, negative feedback from others in the task might make high SA individuals feel rejected, and the loss of points could be associated with impaired performance. Once these key schemas are activated, individuals with high SA often struggle to break these negative cycles.

The current research supports the view of psychological flexibility in general and cognitive flexibility in particular as a resilience mechanism^37^. In line with this view, regulatory flexibility moderated the association between traumatic exposure and PTSD^38^. Cognitive flexibility also moderated the association between trauma exposure and depression, suggesting flexible individuals are less depressed, even after being exposed to trauma^39^. During the COVID-19 Pandemic, parental self-reported psychological flexibility predicted less child and parental stress across time^40,41^ and adaptive coping with the new situation^42^. Taken together, the current study adds to growing research supporting the view that better cognitive flexibility is a transdiagnostic mechanism that fosters better well-being and functioning^22^.

Interestingly, despite the statistical tendency, SA was not significantly associated with social approach behaviors across time. Previous studies examining actual behavior have linked SA to enhanced virtual interpersonal distance^43^, more dating avoidance^44^, and elevated gaze avoidance^45^. Future studies may aim to further test the relationship between updating difficulties and social approach in SA using a larger sample while applying an ecological momentary assessment, which collects data multiple times a day by having its users make immediate reports of certain social behaviors^46^.

The current study has several limitations. First, social approach behaviors were assessed by self-report measures. While this is the common approach when assessing real-life behaviors^47^, we did not examine the underlying purposes of social attendance, which may vary across the wide range of MTurk participants. Future studies may aim to add both objective and nuanced measures, such as in-vivo observations and qualitative descriptions of the motives driving social approach behaviors. Second, the current study was not pre-registered, and the sample includes an analog non-clinical sample. Third, whereas the current study provides valuable longitudinal investigation, Time 2 recruitment included a relatively low sample size. Fourth, due to the relatively small sample size, constructs that are closely related to SA were not part of the statistical model. Fifth, it is possible that the assessment at Time 1 influenced the assessment at Time 2 and perhaps some of the participants became aware of the research hypothesis. Therefore, replicating the current study in a large sample which also includes clinically diagnosed individuals will augment the current model. Sixth, the current study calculated updating using an average score of accuracy, which hinders the variability pattern of updating between participants. A computational modeling approach will provide a better, nuanced understanding of the updating patterns. Lastly, additional research is required to understand the causal relationship between updating and social approach behaviors due to the study’s correlational nature.

To summarize, this longitudinal prospective study is the first to show that a specific deficit in negative-to-positive updating plays a significant role in SA and as well as the involvement in social approach behavior. It may suggest that interventions that improve the ability to positively update behaviors toward other people conducted in a lab setting may positively affect social approach behaviors and facilitate adaptive social functioning in an ever-changing world.

### Supplementary Information

Supplementary Information.

## Data Availability

The dataset and the SEM analysis for this study can be found in the https://osf.io/xhzrc/?view_only=e5e2f557355549f4a90180208a57f7d8.
