# Supplementary material for: Better social reversal learning is associated with a more social approach across time
Source: Sci Rep. 2024 Apr 10;14:8443. doi: 10.1038/s41598-024-58348-5 (PMC11006889; doi:10.1038/s41598-024-58348-5)
Supplement: Supplementary file 1 — Supplementary Information. [file 41598_2024_58348_MOESM1_ESM.docx]

# Supplementary Materials

**Other SEM models**:

Model 1:

Our model considered positive updating and SA at Time 1 as predicting social approach behavior at Time 2. The model displayed poor fit: χ2 (1, N = 126) = 13.429, p < .001. Root-Mean-Square Error of Approximation (RMSEA) = 0.32, the Confirmatory Fit Index (CFI) = 0.33, and the Tucker-Lewis index (TLI) = -3.02. Standardized Root Mean Square Residual (SRMR) = 0.13.

Model 2:

Our model considered SA and the social approach behavior at Time 1 as predicting the social approach behavior at Time 2. The model displayed poor fit: χ2 (1, N = 126) = 8.19, p = .004. Root-Mean-Square Error of Approximation (RMSEA) = 0.24, the Confirmatory Fit Index (CFI) = 0.61, and the Tucker-Lewis index (TLI) = -1.33. Standardized Root Mean Square Residual (SRMR) = 0.09.

Model 3:

Our model considered the social approach behavior at Time 1 as predicting the social approach behavior at Time 2. The model displayed poor fit: χ2 (2, N = 126) = 8.20, p = .017. Root-Mean-Square Error of Approximation (RMSEA) = 0.16, the Confirmatory Fit Index (CFI) = 0.67, and the Tucker-Lewis index (TLI) = -0.00. Standardized Root Mean Square Residual (SRMR) = 0.09.

Model 4^[[1]](#footnote-2)^:

In order to examine the positive updating as a mechanism underlying the change in social approach behavior, our model considered positive updating as a covariate of the latent change score of the social approach behavior at Time 1 and the social approach behavior at Time 2.

The model displayed excellent fit: χ2 (1, N = 126) = 0.239, p = .625. Root-Mean-Square Error of Approximation (RMSEA) = 0.00, the Confirmatory Fit Index (CFI) = 1.00, and the Tucker-Lewis index (TLI) = 1.246. Standardized Root Mean Square Residual (SRMR) = 0.012. We found that negative-to-positive updating was positively associated with the latent change score of the social approach behavior (*β*=.20, *SE*=.24, *p*=.005), suggesting that better negative-to-positive updating is linked to more positive change in the social approach behavior. (See Figure S1).

**Table S1**

Pearson and Spearman correlations of social anxiety severity measures (LSAS and SPIN), accuracies during learning and updating phases, and social approach behavior.

| Variable | 1 | 2 | 3 | 4 | 5 | 6 | 7 |
| --- | --- | --- | --- | --- | --- | --- | --- |
| 1. LSAS | - |  |  |  |  |  |  |
| 2. SPIN | .89^***^ | - |  |  |  |  |  |
| 3. Negative stimulus-outcome learning | .07 | .04 | - |  |  |  |  |
| 4. Positive stimulus-outcome learning | -.11 | -.06 | .03 | - |  |  |  |
| 5. Positive-to-negative updating | .04 | .05 | .57^***^ | .31^***^ | - |  |  |
| 6. Negative-to-positive updating | -.18^***^ | -.16^***^ | .14^*^ | .61^***^ | .29^***^ | - |  |
| 7. Social approach behavior (Time 1) ^a^ | -.11^^^ | -.08^^^ | .04 | .08 | -.03 | .17^*^ | - |
| 8. Social approach behavior (Time 2) ^ab^ | .00 | .04 | .08 | .10 | .03 | .13^***^ | .41^***^ |

^^^*p* < .1. ^**^*p* < .05. ^**^*p* < .01. ^***^*p* < .001

^a^ Spearman correlations

^b^ N=126

LSAS = Liebowitz Social Anxiety Scale. SPIN = Social Phobia Inventory. Negative-outcome associations learning = percentage of avoidance decisions to a negative outcome "people" in the learning phase. Positive-outcome associations learning = percentage of approach decisions to a positive outcome "people" in the learning phase. Positive-to-negative updating = percentage of avoidance decisions to a negative outcome "people" in the updating phase that were associated with a positive outcome during the learning phase. Negative-to-positive updating = percentage of approach decisions to a positive outcome "people" in the updating phase that were associated with a negative outcome during the learning phase. Social approach behavior = number of social events attended, as reported by the participants.

**Figure S1.**

Structural Equation Modeling (SEM) of the role of negative-to-positive updating and social approach behavior in Timr 1 in predicting the change of social approach behavior from Time 1 to Time 2 ^[[2]](#footnote-3)^


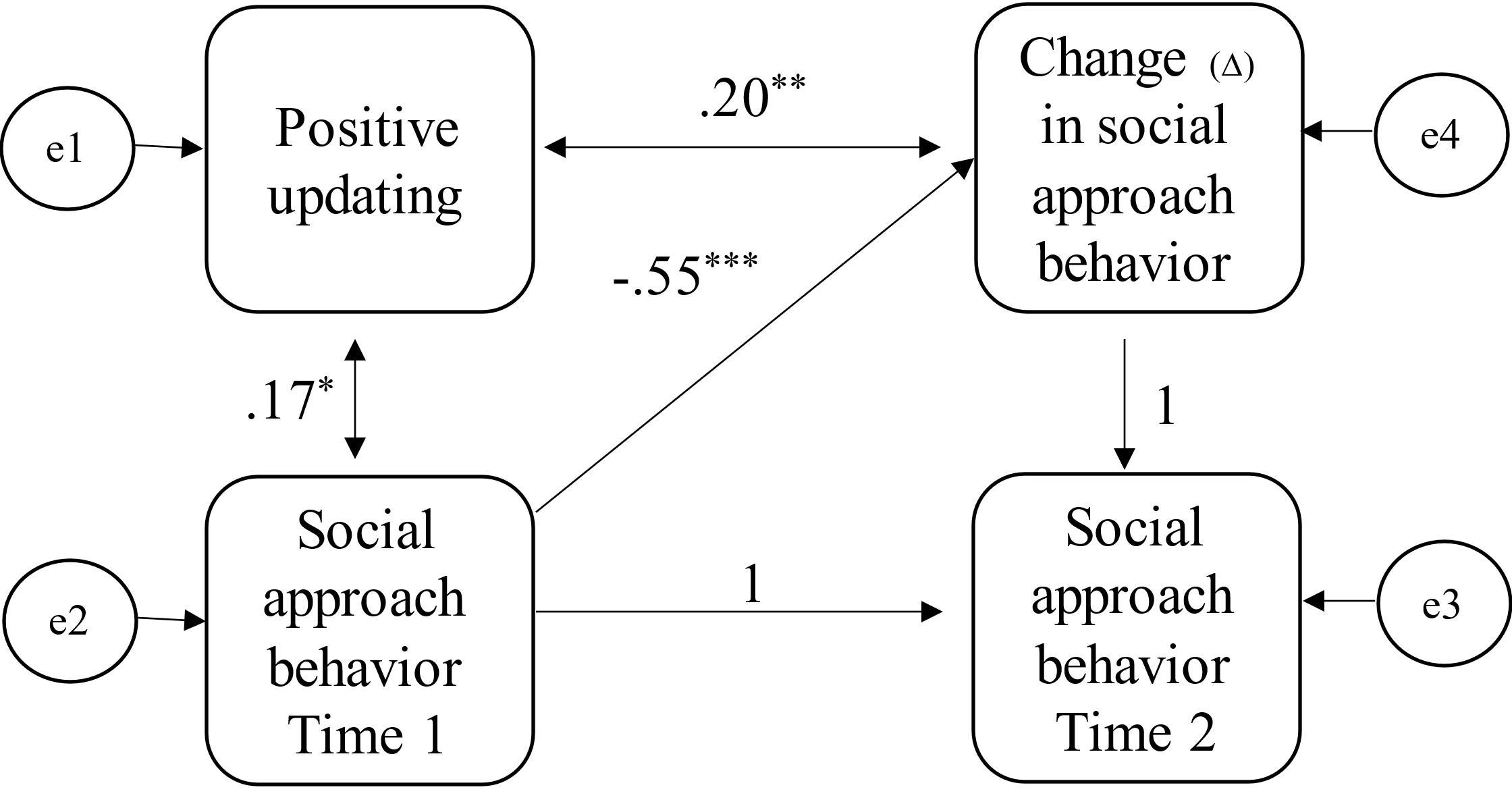


*Note*. ^*^p < .05. ^**^ p < .01. ^***^ p < .001.

Positive updating = accuracy in the Social Reversal Learning Task as measured by the percentage of approach decisions to a positive outcome "people" in the updating phase that were associated with a negative outcome during the learning phase. Social approach behavior = number of social events attended, as reported by the participants.

1. We thank reviewer 2 for suggesting this model. [↑](#footnote-ref-2)
2. We thank reviewer 2 for suggesting this model. [↑](#footnote-ref-3)
